# A case study of the use of verbal reports for talent identification purposes in soccer: A *Messi* affair!

**DOI:** 10.1371/journal.pone.0225033

**Published:** 2019-11-12

**Authors:** Matthew J. Reeves, Allistair P. McRobert, Colin J. Lewis, Simon J. Roberts

**Affiliations:** 1 School of Sport & Health Sciences, University of Central Lancashire, Preston, Lancashire, United Kingdom; 2 The Football Exchange, School of Sport & Exercise Sciences, Liverpool John Moores University, Liverpool, United Kingdom; 3 School of Sport & Exercise Sciences, Liverpool John Moores University, Liverpool, United Kingdom; Nottingham Trent University, UNITED KINGDOM

## Abstract

Using a two-study approach, the main purpose of this case study was to explore the use of a verbal reporting methodology to better understand the thought processes of soccer talent scouts during an in-situ talent identification environment. Study 1 developed a standardized coding-scheme to examine verbal cognitions during a single soccer game. Study 2 then utilized this methodology to examine two full-time recruitment staff trained in the use of concurrent verbal reporting before undertaking a live, in-game task. Participants also participated in a debrief interview following the game. The findings of the two studies suggest that developing a verbal reporting protocol is viable, however when applied in a live-game environment it is problematic. Future research should therefore consider a modified version of this task to further explore the cognitions of scouts whilst observing and identifying potential talent.

## Introduction

In professional sports such as soccer, heads of player recruitment and coaches are constantly striving for the most effective methods of identifying and developing potentially talented youth players [1]. Given the performance advantage a professional soccer organisation can gain over other teams by ‘scouting’ the most talented young players and coupled with the considerable financial rewards potentially on offer, the value of an effective scouting system is evident [2]. From a business perspective, individual players become a valuable human resource [3], which in turn, places considerable importance on the network of talent scouts and recruitment staff who perform the role of identifying and recruiting talented youth players into professional academies. A recent systematic review [4] of talent identification and development in male football identified four broad areas of research interest: 1) task constraints; 2) performer constraints; 3) environmental constraints; and 4) multidimensional analysis. This review, however, identified that there is a larger predisposition for studying developmental aspects of performance, as opposed to identification processes; possibly due to the inherent difficulties associated with identification, especially at younger ages [5]. This is, perhaps, further compounded by the lack of a consensus that defines talent [6, 7, 8].

Researchers interested in talent identification in junior-elite soccer have grappled for many years to develop adequate and objective assessments of talent identification processes in naturalistic and laboratory-based settings [9]. This study, therefore, acts a pragmatic, first step in considering whether a naturalistic approach is feasible and/or appropriate for talent identification purposes. We adopted an exploratory case study design and suggest that results should be treated with appropriate caution given the design utilised.

### Applied talent identification process

Despite the reported methodological constraints synonymous with talent identification research (see [9] for a full review), and as others have already testified [10], identification and selection is a necessary process on a long and winding road to elite performance [11]. Talent scouts act as the on-the-ground face of the clubs that they represent; they are the individuals who often make first contact with potentially talented players. Whilst their primary function is to identify players and pass on information to full-time recruitment staff, they regularly continue to communicate with players and their family during and after a trial period with the club may have taken place [12]. Talent scouts, therefore, play an important role in the decision-making process regarding the players that are recruited to a club; they observe, capture data, and employ subjective judgements based on on-field actions [13].

Despite advancements in technology and the innovation of new multimedia platforms, the ability for academies to collect, collate, and manage data on grassroots junior soccer players is restricted. In most instances, academies collate a range of opinion-based qualitative and quantitative data on individual players that is loosely positioned around the clubs’ recruitment and playing philosophy [14, 15]. Observations are usually, but not always, repeated a number of times before a decision is made about whether or not to recruit a player [12, 14, 15]. Evidence from England, however, suggest that academies are not good at determining or, more precisely, explaining what attributes they are observing when they are trying to identify talented youngsters [14, 15]; a suggestion that is echoed in talent identification and development work elsewhere [5, 16].

Those who undertake scouting roles are, typically, individuals who have spent some considerable time either playing or coaching soccer [15]. However, unlike coaches, talent scouts are not required to possess any formal qualification to undertake the work that they do [17]. The Premier League [17] outlines their recommended qualifications for a range of staff, including coaching and medical, though there is variation between clubs as to how this is operationalised [14]. Coaches working in an academy environment require a range of qualifications. Formal soccer qualifications require coaches to have achieved a defined level of competency in theoretical and practical tasks, and assessments are specific to technical, tactical, strategic, organisational, physiological, and psychological determinants of soccer coaching [18].

This education and training divide between the two groups (i.e. scouts and coaches) is not easily explained especially if one considers the fast-paced, dynamic, and multidimensional nature of soccer. Combined with the speed of player movements, the number of players involved and the subjective nature of visual observations [10, 19], it becomes even less obvious why identification procedures have not received further empirical ecological attention [20]. This ambivalence may be explained by the equivocality surrounding notions of what *talent* [identification] means [in sport] [11] and the confusion and contradictory language which permeates its way through and across the talent development literature (see [21] for a review of psychological terms). Approaching two decades from the publication of the Williams and Reilly [22] model of talent predictors in soccer, talent identification and selection processes continues to rely on apparent subjective (mis)judgements of talent scouts and recruitment personnel.

As noted earlier, little is known about “*what*” talent scouts do, or more importantly “*think*” when identifying and selecting players in either development, or performance domains, during both competition and/or practice in *real time*. Previous soccer talent identification studies using qualitative interview techniques have argued “that coaches regard a player’s speed, play intelligence and attitude toward training and learning the game as criteria they look for when identifying talent” [23]. Whereas others have suggested that the most talented youth soccer players (*i*.*e*. 15–16 years) possess speed, ball control, and an overall desire to succeed [24].

This rather obtuse position is in contrast to a body of well-established research surrounding *talent development* where the influence of the environment in developing the player is considered vital [25]. In support of this Mills and colleagues [26] reported how ten expert development coaches considered discrete psychological factors such as awareness, resilience, goal directed attributes, intelligence, sport-specific attributes, and the environment as fundamental if players were to progress to the professional level. To date, however, there has been little interest in recruitment staff as a participant group for talent identification research, many previous studies have, as already mentioned, tended to utilise coaches [27, 28] despite coaches, arguably, having greater responsibility for player development than identification [12, 14]. Those studies that have included recruitment staff as participants have, so far, used semi-structured interview techniques to elicit the factors affecting the talent identification process from a structural, organisational [12, 14] and philosophical perspective [29].

A potentially useful methodology for addressing this current gap in the talent literature is verbal reports [30]. Since its development by Ericson and Simon [31] (see [32] the use of verbal reports as a technique to elicit the verbalisation of thoughts while performing a task has been widely deployed amongst skilled athletes in exercise settings [33]. Grounded in positivist and empiricist epistemological assumptions, these studies have typically included the use of closed skills from individual sports. For example, in their study of adolescent high-performance golfers, Nicholls and Polman [34] sought to understand acute stress and coping during golf putting performance. Their study demonstrated the appropriateness of concurrent verbal reporting protocols during skill performance to understand how athletes dealt with stress and developed strategies for coping during performance. More recently, Samson *et al*. [35] adopted concurrent verbal reporting for use with distance runners. This study identified how concurrent verbal reporting was appropriate for use during long-distance running and highlighted how data might be used to inform applied psychology support for endurance runners. For example, data suggested all participants found the start of their run difficult, this might highlight a need for sport psychologists to help runners adopt strategies (*e*.*g*. self-talk) that help them overcome the difficulty associated with the early miles of a run. In both studies [34, 35] there was a high level of ecological validity, and athletes were able to verbalise their cognitions appropriately during performance. Despite an abundance of empirical literature originating from the talent in sport domain, a key omission is evidence surrounding “*what*” information talent scouts gather when deployed on scouting assignments or, indeed, evidence of their actual “*thoughts*”.

The aim of this study, therefore, is to present a methodology for depicting concurrent cognitions of talent scouts during part of the talent identification process (*i*.*e*. observing a live game) by means of a verbal reporting protocol. Specifically, it is our intention to consider the feasibility of establishing a standardized reporting protocol for talent identification purposes. Study 1 develops a rigorous coding scheme and player-positional attributes for examining verbal report data. Study 2 utilises the methodology developed in Study 1 to compare the concurrent verbal cognitions of two talent scouts undertaking a “live” talent identification assignment of two junior-elite soccer sides playing against each other.

## Study 1

### Methods

#### Study design and participants

An exploratory case study design was utilised for this study, that allowed the research team adequate flexibility given the multidisciplinary nature of the inquiry [36]. One main advantage of true case study design is its allowance for collaboration between the researcher and participants [37]. The development of our codebook and coding definitions followed a series of systematic and sequential stages. First, a list of specific player attributes identified from a previous talent identification project published and archived elsewhere (*i*.*e*. [38]) was incorporated into a video-based, simulated training and analysis tool. The player attribute categories (*i*.*e*. psychological, technical, physical, and hidden) were subject to content validation by a panel of full-time academy coaches and recruitment staff (n = 3) who were enrolled on an institutional postgraduate degree programme [39]. Panel members ranged in age from 22 to 28 years (M = 25 years; SD = 3).

A 48-match sample of in-game footage of Nike Academy (16-20-year-olds) matches for the 2017 season was used during the study and identified examples of players performing in a number of number of outfield positions (*i*.*e*. central defender, full-back, central midfield, left/right midfield and central wide/attacking player). The Nike Academy was an English football academy funded and administrated by Nike, Inc. until 2017. The academy had a revolving squad of unsigned under-20 players and was run with the intention of helping players find a professional football club. The academy was based at St Georges Park National Football Centre (UK) and the squad was made up of players scouted worldwide and drafted to the squad through the Nike Most Wanted football trials. Full ethical approval was provided by Liverpool John Moores University Ethics Committee (15/EHC/044), and verbal and written informed consent was obtained from the participants and the Nike Academy to use match recordings.

#### Procedure for standardizing player-performance attributes

In this study, we used a reliable and valid procedure to generate video clips of the required attributes for all six outfield playing positions (central defender, fullback, central midfielder, wide midfielder, centre-forward and wide attacker). The standardization process followed the systematic review of 4320 minutes of match footage in order to find video clips that were representative of various outfield positions. All the agreed player film sequences were incorporated into SportsCode Gamebreaker 10.3.1. for editing and reviewing purposes by members of the research team. Training in the use of SportsCode Gamebreaker 10.3.1. was provided by one of the authors (AM) (~3 hours training) who has extensive experience of performance analysis education. In total (n = 15) film sequences were produced for each of the outfield positions (*i*.*e*. central defender, fullbacks, central midfielder, wide midfielder, centre-forward and wide attacker). Each positional sequence contained (n = 10) clips that lasted for approximately 90 seconds each (15.00 minutes total).

#### Validation of coding scheme

To test for attribute acceptance for each position, we asked the participants to watch the video clips and to concurrently verbalise their cognitions from when the video clip started until it ended. Before the beginning of each clip, a black screen presented the name of the playing position and a numbered countdown (3-2-1) was provided, to aid participant visualisation [40]. In addition, a still image with a circle around the player under observation was shown. To achieve acceptable content validity, the observations were recorded and consensus as to whether the attributes were efficiently shown in the player position-specific videos was determined. This approach demonstrated the presence of these attributes and provided the likely language a scout would adopt when thinking about the attribute. In order to confirm the existence of the definitions within each category two members of the research team independently reviewed the recordings and used the following equation to determine inter-observer agreement (IOA): [(Agreements) / (Agreements + Disagreements)] x 100. For example, if there was eight agreements and two disagreements then the equation would be [(8) / (8 + 2)] x 100 = (8/10) x 100 = 80%]. In order to check for observer consistency, the intra-observer reliability (IOR), was established by performing the same test, two weeks after the initial data collection sufficient time for complete memory lapse.

#### Results

The results of this study found that the IOA for central defender was 0.87 (87%); full back 0.80 (80%); central midfielder 0.84 (84%); left/right midfielder 0.80 (80%) and central/wide attacking player 0.86 (86%). The results of the IOR for central defender were 0.83 (83%); full back 0.81 (81%); central midfielder 0.83 (83%); left/right midfielder 0.82 (82%) and central/wide attacking player 0.84 (84%).

## Study 2

### Methods

#### Participants

Following full ethical approval, provided by Liverpool John Moores University Ethics Committee (15/EHC/044), two full-time talent scouts (*i*.*e*. Adam and Ben [pseudonyms]) were purposively sampled from a category one English Premier League academy (see [17], for an overview of academy category status). Adam (44 years) had worked as a talent scout for (17 years) and Ben (26 years) had worked as a talent scout for (3 years). Purposive sampling methods are commonly regarded as suitable for studies where the research team are interested in capturing the best knowledge concerning the research topic and studies which employ content analysis procedures [39]. Prior to commencing the study both Adam and Ben provided written informed consent and were notified that they could withdraw from the study at any time. Gatekeeper consent to undertake video recording was obtained from the club’s academy director as well as the gatekeeper for the opposing team, subsequent informed consent was also obtained from each individual player.

If we are to fully understand the role of the talent scout and their decision-making processes it is important the complexities of the identification process are captured *in situ* before attempting to recreate similar conditions in more controlled, simulated environments [41]. The study was, therefore, conducted at the club’s academy site as the research team were granted permission by the club’s academy director, to observe and record a competitive game between the clubs under 15 team and another junior-elite under 15 team. The game was played mid-week, kick off 1900 hours, on a regulation size (100.5m x 64.0m) artificial 4G pitch, under floodlights with clear weather conditions.

### Procedure

Adam and Ben were trained in concurrent verbal reporting using an adapted version of the instructions outlined by Ericsson and Kirk [42]. This included assigning warm-up exercises such as mental calculations to shape their verbal behaviour. For example, “So that you understand what I mean by think-aloud, let me give you an example. Assume I asked you ‘How much is 127 plus 35?’. Now think-aloud so I can hear how you solve this problem. The participants practised providing verbal reports with feedback provided by members of the research team until level I or II verbal reports was established [43]. Training in concurrent verbal reporting techniques was provided by a member of the research team who has published previously using this procedure in both sport [44] and simulated medical domains [45]. All concurrent verbal report training was conducted on the day of the game to ensure complete understanding of the task requirements.

**Fig 1:**
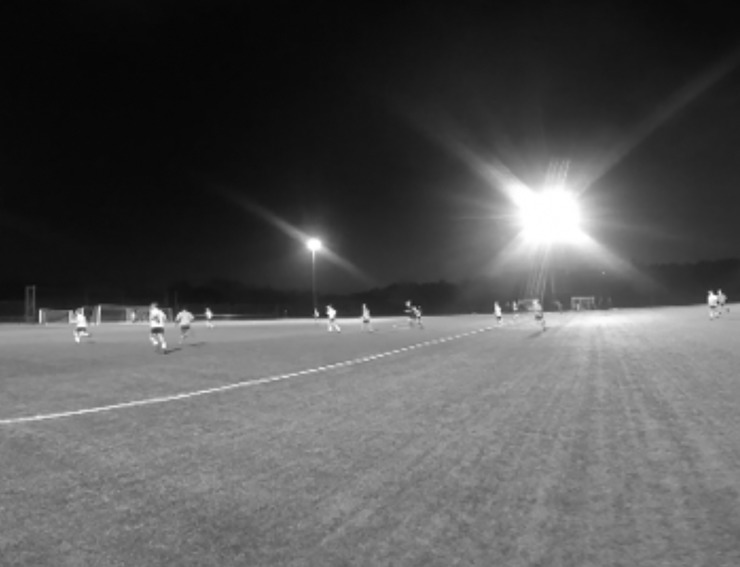
Image of the pitch from Adam’s GoPro camera. ^a^This image has been edited to be black and white to avoid identification of the participating teams through their kit colours.

Prior to the game commencing, a Lavalier microphone and radio transmitter (Sennheiser ew 122-p G3), was connected to a Dictaphone (Olympus WS-853) which was fitted to both participants. Adam and Ben also wore GoPro camera’s (GoPro Hero 5) which were attached to their chest in order to determine whether the team they were scouting were in possession of the football or not. Adam and Ben were instructed to verbalise their thought processes in real-time without self-censoring, or attempting to justify or explain their thoughts, as per the verbal reporting protocol [31]. Each participant took up a position at pitch level on opposite sides of the pitch on the half way line (Fig 1) and engaged in a full 90-minute football game, with the typical 15-minute half-time interval. During the game, Adam and Ben were allocated a research assistant who stood behind them listening for verbal reporting occurring. If either participant stayed quiet for longer than 30-seconds, following verbal reporting protocols, they were prompted by the research assistant to “*think aloud*”.

For the first half of the game, Adam and Ben were asked to focus on the game as a whole. This was intended to represent a scouting assignment where no particular individual had been chosen for observation, and so the scout was responsible for identifying those individuals who they believed to be talented. For the second half, the research team selected two individuals from one team (the team with whom the scouts were not associated) to focus their attention and provide verbal report data.

Following the game Adam and Ben engaged in a debrief and informal semi-structured interview with members of the research team to discuss their thoughts on the verbal reporting protocol, including any difficulties or concerns that they had. This was recorded using a Dictaphone and transcribed away from the academy environment.

### Data analysis

#### Verbal reports

Verbal reports for both participants were transcribed verbatim, generating 15 single-spaced pages of text from 189 minutes and 20 seconds of total recorded audio. Ericsson and Simon [43] outlined analysis procedures for verbal reporting protocols, where they highlighted relevance, consistency, and patterns of verbalisation streams as important. However, due to the exploratory nature of this study, and the broad range of factors likely to be covered by scouts in a dynamic game environment, it was deemed appropriate to conduct line-by-line deductive content analysis (see [46]). Deductive content analysis is often exemplified by cases where researchers wish to code data based on an existing categorisation matrix [46]. Any categorization matrix can be regarded as valid if the categories adequately and accurately captures what was intended [47].

Following transcription, verbal report data were converted to a Notepad text file and imported into Microsoft Excel (2016). Each verbatim statement was then coded using the coding criteria outlined in the codebook generated in Study 1. Each verbal report was then simultaneously and independently coded by a second trained member of the research team using the attribute definitions contained in the codebook. Each concurrent statement was coded using a colour system which was aligned to the attribute presented in Table 1. IOA estimates were conducted using the same method noted above, and suggested that two coders had equivalently coded (70.3%) of the verbalisations. The remaining verbalisations (29.7%) were re-coded by the two raters following a line-by-line debrief and discussion. Following line-by-line deductive content analysis, frequency counts of the individual phrases were imported into the statistical package for the social sciences (SPSS V25) where descriptive statistical analysis procedures were conducted (*i*.*e*. means and standard deviations).

**Table 1:** A detailed overview of the codebook.

Variables	Coding	Example responses
Physical	(1) Acceleration	“Player looks quick, explosive change of pace”
	(2) Agility	“Nice turn, kept possession of the ball despite the defensive pressure”
	(3) Balance	“Looks comfortable on the ball, head up looks balanced”
	(4) Fitness	“Full back is up and down the pitch looks like he can run all day”
	(5) Speed	“Great response, the player really covered the ground didn’t think he/she would get back in time”
	(6) Stamina	“The player in midfield has covered some distance today”
	(7) Jumping reach	“Great leap, really got off the ground to attack the ball”
Psychological	(1) Aggression	“He/she is always putting pressure on the ball”
	(2) Anticipation	“Great play, spots the danger before the cross came over”
	(3) Bravery	“Put his/her body on the line then”
	(4) Composure	“Always looks calm and in control nothing seems to phase him/her”
	(5) Concentration	“Didn’t switch off was alert to the danger”
	(6) Decision-making	“2 v 1 good play–showed real game intelligence”
	(7) Determination	“Can he/she win it, yes well done, first to the ball he/she never gives up”
	(8) Leadership	“Good example by the player let’s see if the others respond”
	(9) Off-the-ball thinking	“Good movement by player, but not necessarily to receive the ball”
	(10) Positioning	“Taken up a great position to allow them to play out”
	(11) Team work	“He/she has put a real shift in for the team”
	(12) Attitude	“Showed a desire there to get back and help”
	(13) Vision	“He/she is scanning”
Technical	(1) First touch	“Poor touch”
	(2) Crossing	“Good delivery out wide into the danger area”
	(3) Corners (delivering)	“Great corner”
	(4) Dribbling/running with the ball	“Can he/she drive–good running with the ball”
	(5) Finishing	“An excellent finish at the near post” / “A poor finish from a good position”
	(6) Free-kicks	“That’s a great ball into a dangerous area”
	(7) Heading	“They’ve got up well, there”
	(8) Long-range shooting	“An excellent effort from distance, there”
	(9) Long-throw ins	“Need to be careful of his/her long throw in this position”
	(10) Passing accuracy	“Fantastic range and accuracy of passing”
	(11) Marking	“Don’t let him play it”
	(12) Penalty taking	“He/she has approached that calmly and sent the keeper the wrong way”
	(13) Tackling	“Luckily they’ve got that tackle timed perfectly”
	(14) 1 v 1	“If they can get the ball out wide they’ve got a 1 v 1”
	(15) Technique-under pressure	“Excellent turn to get out of a difficult position, there”
Hidden	(1) Adaptability	“The players have changed to his style of play”
	(2) Consistency	“His/her consistency is a great attribute”
	(3) Versatility	“He/she’s switched into the [alternative position] role seamlessly”
	(4) Important matches	“This is a game where he/she will shine”
	(5) Coachability	“They’re always listening to what the coach is saying, no matter what”
	(6) Communication	“Good talking between units”
	(7) Flair	“An amazing bit of skill to get away from the defender”
	(8) Creativity	“That’s a very clever decision”
Tone of statement	(1) Positive	“Great first-touch”
	(2) Negative	“Awful, there was no way that ball was ever going to reach the wide-player”
	(3) Neutral	“Can he/she play”
	(4) Unknown	“There is a lot more that’s not known at the moment”

#### Informal debrief

The informal debrief data were transcribed line-by-line and content analysis with inductive reasoning was conducted to develop themes and a process of continual examination and comparison was performed. Following hermeneutic procedures provided by Thomas and Pollio [48], information-rich verbalisations were identified as meaning units, which were subsequently grouped into sub-themes. Verbalisations were pieces of coded text that related to an attribute and ranged in the number of words they contained. This process was initially completed by two (MJR & SR) of the research team before being shared with the remaining two team members (AM & CL) to consider trustworthiness surrounding interpretation of data.

## Results

### Descriptive analysis–verbal reports

The sample text references included a total of 11,696 words. There were 331 psychological attribute verbalisations coded (M = 9.9 words, SD = 14.09); 316 were coded as technical attribute verbalisations (M = 22.57 words, SD = 30.36) and 56 were coded as physicality verbalisations (M = 8 words, SD = 5.54). The tone of verbalisations were mostly neutral (48%). Positive verbalisations were coded 27% of the time, with negative accounting for 17%, and unknown 8%. For a full breakdown of the findings please refer to Table 2.

**Table 2:** Descriptive analysis of verbalisations.

		Frequency	Percent of attribute verbalisations	Percent of all verbalisations
Psychological attribute verbalisations	Anticipation	16	4.8	2.2
	Concentration	6	1.8	0.8
	Decision-making	158	47.7	21.7
	Determination	18	5.4	2.5
	Leadership	2	0.6	0.3
	Off-the-ball thinking	43	13.0	5.9
	Positioning	40	12.1	5.5
	Team work	2	0.6	0.3
	Attitude	38	11.5	5.2
	Vision	8	2.4	1.1
	**TOTAL**	**331**	**100**	**45.5**
Technical attribute verbalisations	First touch	73	23.1	10.0
	Crossing	26	8.2	3.6
	Corners (delivering)	2	0.6	0.3
	Dribbling/running with the ball	38	12.0	5.2
	Finishing	12	3.8	1.7
	Free-kicks (delivering)	2	0.6	0.3
	Heading	6	1.9	0.8
	Shooting	12	3.8	1.7
	Long-throw ins	10	3.2	1.4
	Passing accuracy	104	32.9	14.3
	Marking	4	1.3	0.6
	Tackling	6	1.9	0.8
	Technique under pressure	19	6.0	2.6
	Penalty taking	2	0.6	0.3
	**TOTAL**	**316**	**100**	**43.5**
Physical attribute verbalisations	Acceleration	2	3.6	0.3
	Agility	16	28.6	2.2
	Fitness	8	14.3	1.1
	Speed	12	21.4	1.7
	Stamina	4	7.1	0.6
	Strength	12	21.4	1.7
	Jumping reach	2	3.6	0.3
	**TOTAL**	**56**	**100.0**	**7.7**
Hidden attribute verbalisations	Communication	24	100	3.3
	**TOTAL**	**24**	**100**	**3.3**

### Psychological attribute verbalisations

The highest frequency (n = 158) of coded verbalisations related to *decision-making* thoughts (47.7%). For example, in the first half of the game Adam stated: “*Look to play*, *look to be positive good decision didn’t force it*” and “*Number four has it he’s a threat*, *can he get on it in midfield*, *no wrong way–poor decision*”. The second most prominent verbalisation was *off-the-ball thinking* (13%) followed by *positioning* (12.1%). For example, in the first-half when the team Adam was scouting were not in possession of the football he said, “*Got to see the danger on the weak side*, *he needs to drop in and get in position–poor play he was ball watching*.” Adam and Ben also made a number of comments (11.5%) which were coded as *attitude* verbalisations. For example, following a mistake on the ball Adam commented “*Poor play a lack of quality maybe too quick*, *now he needs to work hard and recover–good recovery there from the right back*”.

### Technical attribute verbalisations

The participants mentioned a number of technical attributes during the game, but the most prominent thoughts related to *passing accuracy* (32.9%), *first-touch* (23.1%) and *dribbling/running with the ball* (12.0%). Typical positive examples from Ben included “*Good pass from [blinded]*. *Good ball*”, a negative example “*Bad touch from [blinded]*, *should have done better*” and a neutral example included “*Can he travel*, *can he travel*. *Can he go forward*”. Examples from Adam included “*Great ball*, *good delivery well done*”, and “*Hold*, *hold*, *hold*. *Keep hold of it*. *Keep hold of it*” and “*Keep the ball*, *attack him*, *good*. *Keep going forward*, *set up the cross*.”

### Physical attribute verbalisations

The talent scouts mentioned 56 thoughts that related specifically to physical attributes. The scouts commented positively on players agility (28.6%), speed (21.4%) and strength (21.4%). For example, Ben stated, “*great turn and change of direction–don’t stop drive, drive*” and “*That lad on the ball is quick–left side*” and “*Good strength through the middle*”.

### Hidden attribute verbalisations

The only hidden attribute that participants mentioned was communication (100%). For example, Adam noted “*good talking from the skipper* [team captain] *there*” and “*he’s spending a lot of time talking*”.

### Informal interview

During the debrief with Adam and Ben, both indicated that the task was ‘extremely difficult’, more so than they had imagined it would be during the training. Following further discussion, participants identified that ‘things moved really quickly’ and they were ‘barely able to do more than commentate’. Indeed, the fast-paced nature of the game and the low-level of detail provided in the verbalisations suggest that the cognitive load was high for this particular task. That is, there was a lot of visual information for Adam and Ben to observe, synthesise, and verbalise before the game had already progressed. “*It made keeping up with the game really hard…I felt like I’d not finished [verbalising] but I needed to move on to the next bit*.”

The informal debrief with both participants was conducted approximately five minutes following the conclusion of the game. The debrief was short (19 minutes in total) but indicated the need for the research team to consider different approaches to examining the cognitive processes and strategies adopted by those responsible for talent identification in junior-elite football. When we asked Adam and Ben to reflect on the players that they were tasked with scouting in the second half it was interesting to note that both disagreed with the player attributes and both disagreed regarding the recruitment philosophy of their own club. Interestingly, the recruitment philosophy was visible in the interview room–a vinyl graphic occupying approximately two-thirds of a wall–when Adam and Ben were asked how accurate their thoughts and observations were in relation to this, they responded by further disagreeing with their previous verbalisations and aligning their responses to factors highlighted in the vinyl graphic. Such dissonance between philosophies and on-the-ground practice have been reported in previous studies [12, 14].

## Discussion

The aim of this case study was to explore the use of a verbal reporting methodology to better understand the thought processes of soccer scouts during a live junior-elite soccer game. Study 1 developed a rigorous standardized verbal report coding scheme to be used in the analysis of verbal reports. The standardized coding form was created with the aim of providing an objective view of talent identification attributes that could be used in a practical setting. The content validation of the coding system suggests it is a versatile analysis tool which could be used to inform future talent identification studies or the training of talent scouts.

In the second study, talent scouts’ thoughts were captured during a live game utilising a verbal reporting protocol [43] and verbalisations were analysed using deductive content analysis. To our knowledge this is the first study to attempt to capture the thought processes of talent scouts using concurrent verbal reports despite this methodology featuring prominently in existing cognitive control accounts of skilled athletic performance [49]. This study, therefore, acts as a preliminary first step in the applied body of work in this area. Findings suggest that while the live-game observation yields high ecological validity, the dynamic nature of football creates too many variables for cognitions to be accurately verbalised due to time-pressures associated with the speed of the game. Participants, whilst attempting to do so, found themselves commentating as opposed to fully verbalising the cognitions of what they were seeing and how they were making sense of it and so some caution is required when interpreting these findings. Data suggested that Adam and Ben did not alter what or how they undertook scouting, regardless of the task focus (*i*.*e*. full team versus specific players). Indeed, there was no difference in the tone or number of verbalisations between the two tasks. When focusing on a specific player(s) Adam and Ben’s thought processes appeared to remain game focussed when their intended focus (*i*.*e*. a specific player) was out of possession and/or not particularly involved within a phase of play.

### Psychological attributes

The most frequent perceptual-cognitive thoughts were coded as *decision-making* (*n* = 158) *off-the-ball thinking* (*n =* 43), and *positioning* (*n* = 40). The most frequent psychological attribute thoughts was *attitude* (*n* = 38). Perceptual-cognitive skills such as *decision-making*, and *off-the-ball* thinking are repeatedly reported to be advantageous in team sports and soccer specifically [50, 51]. Decision-making ability in a team sports such as soccer is commonly defined as the appropriateness of a decision, preceding a suitable action and is relative to the game context and specific interactions which occur between players of the same team and the opposition [52]. For example: “*If my direct defensive opponent is far away from me, then I will shoot; or, if he closes me down, then I will do a step-over and drive past him*” [53]. This ability to carry out two concurrent skills (*i*.*e*. dribbling the ball while scanning the pitch for the opposition or teammates) is considered an important attribute for performance in team sports [53]. The talent scouts in our study were coded when they explicitly commented on the players’ on-the-ball decisions, however, like others [52] the quality of the decision was difficult to assess, and as we did not explicitly assess whether the player decisions were ‘appropriate’ or ‘inappropriate’ we recommend that further work is conducted in this area. The high number of off-the-ball verbalisations is an interesting one especially when the majority of these were captured during the first-half when the scouts were requested to observe the whole game and not focus on a specific player or position. When we analysed the GoPro footage and cross-referenced the off-the-ball verbalisations it was apparent they were verbally reporting while still tracking the ball and, therefore, processing large amounts of information. It would appear the scouts were using effective visual search strategies, scanning the whole pitch and filtering lots of contextual information very quickly. At this stage we acknowledge that this is pure conjecture and requires more detailed experimental analysis in simulated conditions. This is, however, an interesting supposition as eye fixations are known to be one of the pre-requisites for superior performance in sport and other areas [54]. At this stage we are not aware of any eye fixation work that has been conducted with talent scouts or recruitment staff and although this is pure speculation at the moment, it may be worthy of further investigation. The procedural knowledge involved in the interpretation of a specific situation and the ability to be/or to get in the right place at the right time (*i*.*e*. positioning) is known to be a prerequisite for excellence in team sport [55]. *Positioning* is, however, dependent on systems of play, for example in a 3-5-2 system, the full-back or wide player (dependent on whether the team is in possession of the ball or not) may need to act as an attacker or defender. Future talent studies, therefore, may need to consider team formations and player positions *a priori*.

### Technical attributes

The most frequent technical thoughts were attributed to *passing accuracy* (*n* = 104), *first-touch* (*n* = 73), and *dribbling/running with the ball* (*n =* 38). These findings are consistent with previous talent studies in soccer such as Larkin & O’Conner [29], who also reported first-touch and striking the ball as important. A technique was defined as the ability to carry out a solitary action with minimal cognitive decision-making. Passing accuracy (*i*.*e*. appropriate speed and angle) is considered an important technical attribute, especially for teams with a ball possession style of play. Despite a positive association between possession of ball time and team success [56] some caution is required as ball possession is multifaceted by extenuating factors such as playing style, quality of the opposition and the score of the match [57, 58, 59].

### Physiological attributes

The most commonly coded physiological statement was *agility* (*n* = 16), however, physiological verbalisations were considerably lower than psychological and technical. Indeed, of the 727 verbalisations coded, only 56 (7.7%) were physical or physiological. There is a long history of talent-related literature suggesting a pre-disposition, or bias, toward physical and physiological factors associated with talent [6, 60, 61]. Indeed, much of the literature pertaining to relative age effect (RAE) has indicated that junior-players are more likely to be selected due to factors significantly affected by relative age [62, 63, 64]. The two other highest coded attributes were *speed* (*n* = 12) and *strength* (*n* = 12). Collectively, these three attributes have been considered in a number of previous studies [65, 66, 67] and their findings now considered best evidence in terms of the importance for talent identification, development, and monitoring purposes [68].

### Hidden attributes

*Communication* (*n* = 24) was the only statement that was coded from the hidden attribute category. Similarly, this was the lowest coded attribute category, with only 24 (3.3%) verbalisations.

### Strengths and limitations

Strengths of this study include a novel, two-study methodological approach to capturing scouts’ concurrent cognitions during in an *in-situ* environment. The study also captures follow-up qualitative data in an attempt to understand any holistic or philosophical differences regarding talent identification practice. A more comprehensive study design should incorporate and include talent scouts from different academies working colligatively, however, professional soccer clubs and their academies are not renowned for working in partnership and instead tend to be recondite about recruitment practice(s) [15]. This study, therefore, offers a unique insight into talent scouts’ “*thoughts*” on working in a professional soccer environment.

Despite these strengths, the study contains several shortcomings that need to be considered by researchers in talent identification. Firstly, although independent IOA estimates were acceptable, some of the coding constructs (*e*.*g*. *decision-making*, *technique-under-pressure*, and *off-the-ball thinking*) required interpretation from the research team. For example, it was difficult to distinguish between whether a decision-making thought reflected an on-the-ball (*i*.*e*. a skill or technique) or off-the-ball action. It is our contention that further validation of these constructs is required. Secondly, a small purposive sample of English talent scouts was used and although this sample is in-line with other verbal reporting studies [69] we acknowledge this as a less than representative sample. However, professional soccer clubs in England are notoriously secretive about their recruitment procedures and practices, and as other researchers can testify gaining access and acceptance in these environments can be extremely difficult [70]. Thirdly, as the qualitative data alludes to “*thinking out loud*” for the duration of a full 90-minute game was mentally draining for the participants and difficult. This may have impacted on how Adam and Ben undertook the two scouting tasks (*i*.*e*. whole team identification versus observing specific players). Indeed, both may have, potentially, been mentally fatigued following the first half and unable to differentiate between the tasks adequately.

Despite the current methodological shortfalls, modified versions of the task presented may offer future avenues for research in this area. Specifically, future research may be better to adopt a more controlled, lab-based, environment to examine the cognitive thought processes of scouts, and recruitment staff in more using larger sample sizes. Following Eccles and Arsal [71] we would also encourage a more detailed qualitative component to future studies that captures the nuances of how club recruitment philosophy influences the decisions made by staff responsible for this area of work for the football club. Finally, examining eye fixation would be an interesting development in this area so it is possible to determine where a scout is looking during a game. Eventually, this research might generate more accurate and reliable information for practitioners and researchers interested in understanding the complexities of the talent identification process.
